# Autonomic Function in Patients With Parkinson’s Disease: From Rest to Exercise

**DOI:** 10.3389/fphys.2021.626640

**Published:** 2021-03-19

**Authors:** Jeann L. Sabino-Carvalho, James P. Fisher, Lauro C. Vianna

**Affiliations:** ^1^NeuroV̇ASQ̇ – Integrative Physiology Laboratory, Faculty of Physical Education, University of Brasília, Brasília, Brazil; ^2^Manaaki Mānawa – The Centre for Heart Research, Department of Physiology, Faculty of Medical and Health Sciences, The University of Auckland, Auckland, New Zealand; ^3^Graduate Program in Medical Sciences, Faculty of Medicine, University of Brasília, Brasília, Brazil

**Keywords:** exercise, dorsal motor nucleus of the vagus nerve, parasympathetic activity, sympathetic activity, blood pressure

## Abstract

Parkinson’s disease (PD) is a common neurodegenerative disorder classically characterized by symptoms of motor impairment (e.g., tremor and rigidity), but also presenting with important non-motor impairments. There is evidence for the reduced activity of both the parasympathetic and sympathetic limbs of the autonomic nervous system at rest in PD. Moreover, inappropriate autonomic adjustments accompany exercise, which can lead to inadequate hemodynamic responses, the failure to match the metabolic demands of working skeletal muscle and exercise intolerance. The underlying mechanisms remain unclear, but relevant alterations in several discrete central regions (e.g., dorsal motor nucleus of the vagus nerve, intermediolateral cell column) have been identified. Herein, we critically evaluate the clinically significant and complex associations between the autonomic dysfunction, fatigue and exercise capacity in PD.

## Introduction

Parkinson’s disease (PD) is currently the second most common neurodegenerative disorder (Alves et al., 2008; Rossi et al., 2018) and the worldwide prevalence is growing as age and life expectancy increases (Rossi et al., 2018). The disease is well-characterized by a dysfunction of dopamine-producing neurons in the substantia nigra pars compacta and was first described more than 200 years ago (Parkinson, 2002). This dopamine deficiency leads the classical motor dysfunctions (bradykinesia, rigidity, and resting tremor) featuring the disease (Braak and Braak, 2000). In addition, PD may include several non-motor impairments, including autonomic and cardiovascular dysfunction (Gallagher et al., 2010; Goldstein, 2014; Merola et al., 2018; Sabino-Carvalho et al., 2021). Furthermore, some aspects of the autonomic dysfunction can precede the motor dysfunction by more than a decade (Pont-Sunyer et al., 2015). Therefore, early detection of autonomic dysfunction may allow an early diagnosis (Kaufmann et al., 2004, 2017) and seem to make an important contribution to the PD pathophysiology (Goldstein, 2014; Greene, 2014).

Sympathetic and parasympathetic branches of the autonomic nervous system are crucial for homeostasis control (Billman, 2020) and are essential for ensuring that the appropriate cardiovascular and hemodynamic adjustments to exercise occur, such that the metabolic demands of working skeletal muscle are met. Conversely, autonomic dysfunction is associated with fatigue, impaired exercise capacity, and poor quality of life in several patient populations, including PD (Chaudhuri and Behan, 2004; Gallagher et al., 2010; Nakamura et al., 2011; Vianna and Fisher, 2019; Pechstein et al., 2020). The present mini-review will critically evaluate the evidence for parasympathetic and sympathetic dysfunction in PD both at rest and during exercise, explore how such autonomic dysfunction may affect exercise capacity, and explore the therapeutic potential of exercise training.

### Parasympathetic Dysfunction in PD

The parasympathetic preganglionic neurons are situated in the nucleus ambiguous (NA) and dorsal motor nucleus of the vagus nerve (DMV). The axons travel within the vagus nerve (tenth cranial nerve) and synapse at postganglionic neurons, such as those located at the cardiac plexus. While the NA contains vagal preganglionic neurons that inhibit the heart rate (Machado and Brody, 1988), the activity of the DMV vagal preganglionic neurons are responsible for tonic parasympathetic control of ventricular excitability (Machhada et al., 2015). Cardiac autonomic parasympathetic neurotransmission is accomplished through the release of acetylcholine and other substances onto receptors located in atrial and ventricular myocardium, modulating the chronotropic, inotropic and dromotropic properties of the heart (Degeest et al., 1964; Coote, 2013).

In patients with PD, a common pathological hallmark is the presence of Lewy bodies. These abnormal aggregates of α-synuclein protein are widely distributed in the hypothalamus, sympathetic [intermediolateral cell column (IML) and sympathetic ganglia] and parasympathetic centers (DMV, NA, and sacral parasympathetic nuclei), and may disrupt the central components of autonomic reflex arc involved in autonomic regulation (Braak and Braak, 2000; Del Tredici and Braak, 2016; Nakamura et al., 2016; Wang et al., 2020). Human studies and animal models of PD provide quantitative evidence for damage to brainstem parasympathetic neurons and in vagus nerves (Beach et al., 2010; Machhada et al., 2015; Walter et al., 2018). Post mortem studies of patients with PD have shown the presence of α-synuclein protein in the vagus nerve (Beach et al., 2010) and *in vivo* studies have reported significantly smaller vagus nerve axons in patients with PD (Pelz et al., 2018; Walter et al., 2018). With regards to animal models of PD, a reduced activity of DMV neurons is reported (Machhada et al., 2015). In fact, the DMV has been hypothesized by Braak et al. (2003) as being one of the first areas to be affected during the pathological progression of PD in humans, although this is not a universal finding (Kalaitzakis et al., 2008).

Heart rate variability (HRV) derived estimates of cardiac parasympathetic activity have been observed to be reduced in patients with PD (Kallio et al., 2000; Buob et al., 2010; Sabino-Carvalho et al., 2018; Li et al., 2020) independent of the measurement duration/experimental circumstance. However, this finding is not unanimous (Oka et al., 2011; Kiyono et al., 2012; Vianna et al., 2016). Intriguingly, Alonso et al. (2015) demonstrated that the lower the root mean square of successive differences in N–N intervals (RMSSD, a time domain marker of parasympathetic activity) and the standard deviation of N–N intervals (SDNN, the standard deviation of interbeat intervals), the greater the chances of PD developing during an 18-year follow-up period. However, Alonso’s findings should be interpreted with caution, as the measurement duration (i.e., 2-min) is considered suboptimal for this purpose by the Task Force of the European Society of Cardiology and the North American Society of Pacing and Electrophysiology (i.e., ≥5-min) (Task Force, 1996). Altogether, these findings may suggest that aggregates of α-synuclein protein in the brainstem parasympathetic neurons might play a role in HRV alterations observed in patients with PD, however, this possibility remains to be tested.

### Sympathetic Dysfunction in PD

Sympathetic preganglionic cell bodies are located in the IML of the spinal cord and the preganglionic fibers directed to the heart synapse at the stellate ganglion and upper thoracic ganglia (T1–T5). Postganglionic sympathetic fibers synapse in the heart (at the sinoatrial node, atrioventricular node, atria, and ventricles) and in the vasculature. The sympathetic preganglionic neurons in the IML receive strong excitatory drive from neurons of the rostral ventrolateral medulla (RVLM) (Dampney, 1994). Sympathetic preganglionic neurons at the IML also receive direct excitatory inputs from other regions of the central nervous system including the ventromedial medulla, caudal raphe nuclei, A5 noradrenergic cell group of the caudal ventrolateral pons, and the paraventricular hypothalamic nucleus (Dampney, 1994; Dampney et al., 2003). The sympathetic preganglionic neurotransmission is accomplished by the release of acetylcholine, whereas postganglionic neurons release norepinephrine (and other co-transmitters) onto α- and β-adrenergic receptors modulating both chronotropic and inotropic properties of the heart and the peripheral resistance in the vasculature (Drew and Whiting, 1979).

Orthostatic hypotension (OH) affects approximately 50% of the patients with PD and the prevalence may be higher when considering asymptomatic OH (Palma et al., 2015). Noteworthy, OH is often used as a clinical indicator of sympathetic nervous system dysfunction (Goldstein et al., 2015; Kaufmann et al., 2020; Palma and Kaufmann, 2020). Central lesions in the brainstem are thought to mediate the sympathetic dysfunction in PD (Metzger and Emborg, 2019), as showed by Braak and colleagues through analysis of the regional distribution of α-synuclein immunoreactive structures in the brain of 110 subjects (Braak et al., 2003, 2004; Braak and Del Tredici, 2010). This proposed model suggests that the PD process begins in the lower brainstem in the DMV, as well as in the anterior olfactory structures. Thereafter, the disease rostrally ascends from the DMV through regions of the medulla, pontine tegmentum, midbrain, and basal forebrain, reaching the cerebral cortex. Of note, as PD progresses upward from the brainstem, both the severity of the lesions and clinical manifestations of the PD symptoms increase (Braak et al., 2004). The mechanisms and precise neuronal profile damage responsible for the sympathetic dysregulation are not fully understood. However, studies have indicated that the abnormal aggregates of α-synuclein protein distributed in the IML and sympathetic ganglia contributes to the sympathetic dysfunction (Braak and Braak, 2000; Del Tredici and Braak, 2016; Nakamura et al., 2016; Wang et al., 2020). Moreover, post mortem analyses of patients with PD also showed a selective loss of C1 and C3 neurons (Gai et al., 1993) providing quantitative evidence for brainstem damage of sympathetic neurons. Cardiac sympathetic denervation and lower norepinephrine spillover are also concomitant with the central lesions in patients with PD (Goldstein et al., 2003; Nakamura et al., 2014).

The microneurography technique provides a valuable tool for direct recording of muscle sympathetic nerve activity (MSNA) in humans (Carter, 2019). Shindo et al. (2003) demonstrated that resting MSNA was similar between patients with PD and control subjects. However, a negative association was observed between MSNA and both age and disease duration in PD, reflecting the impact of PD progression on sympathetic dysfunction (Shindo et al., 2003). Nevertheless, the inherent characteristics of PD (i.e., resting tremor and involuntary movements) makes it challenging to obtain and maintain MSNA recordings in this population, and thus only a few studies have attempted this (Shindo et al., 2003, 2005; Sverrisdóttir et al., 2014) and unfortunately not all compared their results with control subjects (Shindo et al., 2005; Sverrisdóttir et al., 2014). Sympathetic postganglionic neuronal function in the heart has been assessed in PD (Rascol and Schelosky, 2009). A myocardial concentration of metaiodobenzylguanidine (MIBG), a physical analog of noradrenaline that is transported into sympathetic terminals, has been used as a biomarker of cardiac sympathetic denervation in PD. Indeed, abnormal MIBG uptake has been reported in patients with PD both with (Nakamura et al., 2014) and without (Oka et al., 2011) OH. However, the cost and invasiveness of the MIBG technique have limited its use in the experimental assessment of cardiac sympathetic activity. Given this, alternative approaches, such as the analysis HRV, may provide important advancements in our understanding of sympathetic dysfunction in PD.

**FIGURE 1:**
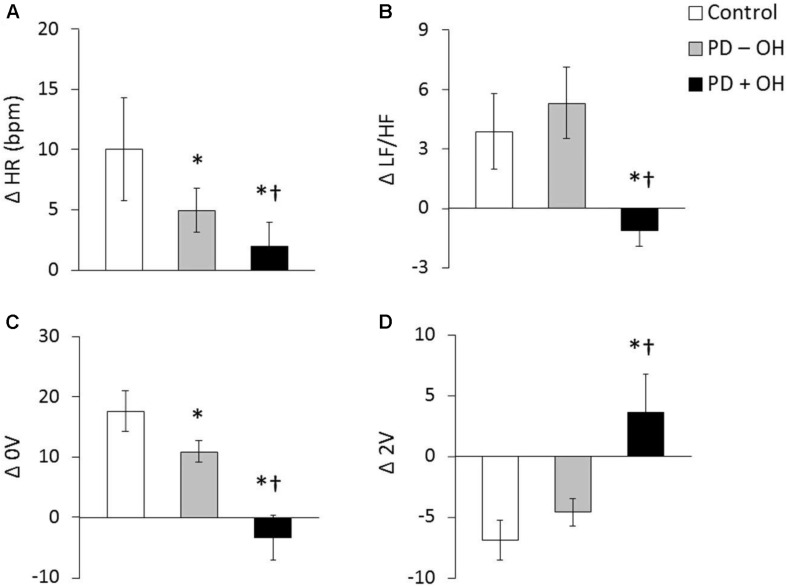
Heart rate and LF/HF ratio in response to active standing in controls and in patients with PDwith (PD + OH) and without (PD – OH) orthostatic hypotension (**A** and **B**, respectively). Non-variable (0V – a proxy of sympathetic activity, **C**) and very variable (2V – a proxy of parasympathetic activity, **D**) category of symbolic dynamics analyses of heart rate variability. *P < 0.05 compared with control group. ^†^*P* < 0.05 compared with PD – OH group. Mean data from Vianna et al. (2016).

Despite the fact that most indices derived from HRV primarily reflect vagal function, the joint analysis of the low frequency (LF) component, expressed in normalized units, and the LF/HF ratio, has been suggested to be an acceptable marker of sympathetic modulation (Malliani et al., 1994) although this has been challenged (Parati et al., 2006). In this regard, a decreased LF (Li et al., 2020), LF/HF ratio at rest (Oka et al., 2011; Sabino-Carvalho et al., 2020) and a blunted increase in LF/HF ratio during tilt-table testing (Friedrich et al., 2010), has been identified in patients with PD compared to controls. However, this result is not unanimous, and other studies have not observed LF/HF ratio to be decreased at rest in PD (Bouhaddi et al., 2004; Friedrich et al., 2008; Vianna et al., 2016; Sabino-Carvalho et al., 2018). Notably, in patients with PD and OH both a lower resting LF/HF (Barbic et al., 2007) and a blunted LF/HF increase during an active standing test (Vianna et al., 2016) has been observed. This blunted response was also observed through analysis of the sympathetic component of HRV using symbolic analysis. Whereas, those patients without OH demonstrated only an attenuated response during an active standing test compared to controls (Figure 1, Vianna et al., 2016). Taken together, these data highlight the presentation of sympathetic dysfunction in PD and suggest that the co-existence of OH, where a reduced vasoconstriction is thought to be a pathophysiological reason (van Wijnen et al., 2018), indicates a more severe condition.

### Cardiovascular Responses to Exercise

During exercise, several neural mechanisms work in concert to precisely control cardiovascular and hemodynamic responses (Fisher et al., 2015). In neurodegenerative disorders, such as multiple system atrophy and pure autonomic failure, an abnormal cardiovascular responses to exercise is often observed (Low et al., 2012). These patients commonly have a blunted BP increase during isometric exercise (Khurana and Setty, 1996), and may even demonstrate a marked exercise-induced hypotension during dynamic exercise (Akinola et al., 2001). Considering the importance of the sympathetic contribution to the BP response to exercise, studies have suggested that sympathetic nerve activity is reduced during exercise in these patients (Kachi et al., 1988; Dotson et al., 1990; Donadio et al., 2010). Overall, this scenario is quite similar in PD, especially in those patients with OH (Marshall et al., 1961), where parasympathetic and sympathetic dysfunction together may interact to cause the abnormal cardiovascular responses to exercise.

**FIGURE 2:**
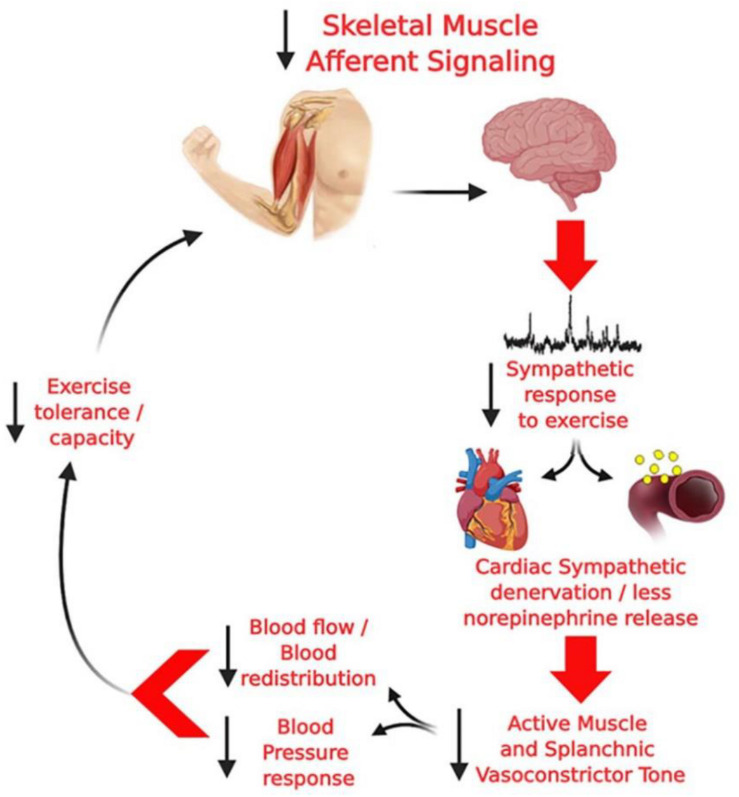
Hypothetical model from Sabino-Carvalho and Vianna (2020) of the potential deleterious consequences of altered group III and IV skeletal muscle afferent activity in PD. Diminished skeletal muscle afferent signaling, together with central degeneration in PD, can result in a blunted sympathetic vasoconstriction leading to a blunted pressor response and, consequently, to lower increase blood flow to the active muscle, which may lead to metabolic distress, tissue/brain hypoperfusion, fatigue, cardiac autonomic imbalance, and an impaired exercise capacity.

The first evidence for an attenuated cardiovascular response to exercise in PD was provided in the late 80’s by Sachs et al. (1985), Ludin et al. (1987), and Turkka et al. (1987) who demonstrated a blunted BP increase during isometric handgrip exercise. This finding was recently reproduced in dynamic exercise involving a large muscle mass (Reuter et al., 1999; Werner et al., 2006; DiFrancisco-Donoghue et al., 2009; Kanegusuku et al., 2016), resistance exercise (Miyasato et al., 2018) and isometric handgrip exercise (Sabino-Carvalho et al., 2018). Indeed, an inability to increase sympathetic activity to important compliant regions (i.e., splanchnic circulation), may impair the blood flow redistribution to the contracting muscles during exercise in PD (Sabino-Carvalho and Vianna, 2020) and, therefore, the reduced vasoconstriction in vascular beds will directly affect BP response to exercise. Consequently these impaired responses might contributed to metabolic distress, tissue/brain hypoperfusion, fatigue, cardiac autonomic dysfunction, and an lower exercise capacity, however, this hypothesis remains to be tested (Figure 2).

**FIGURE 3:**
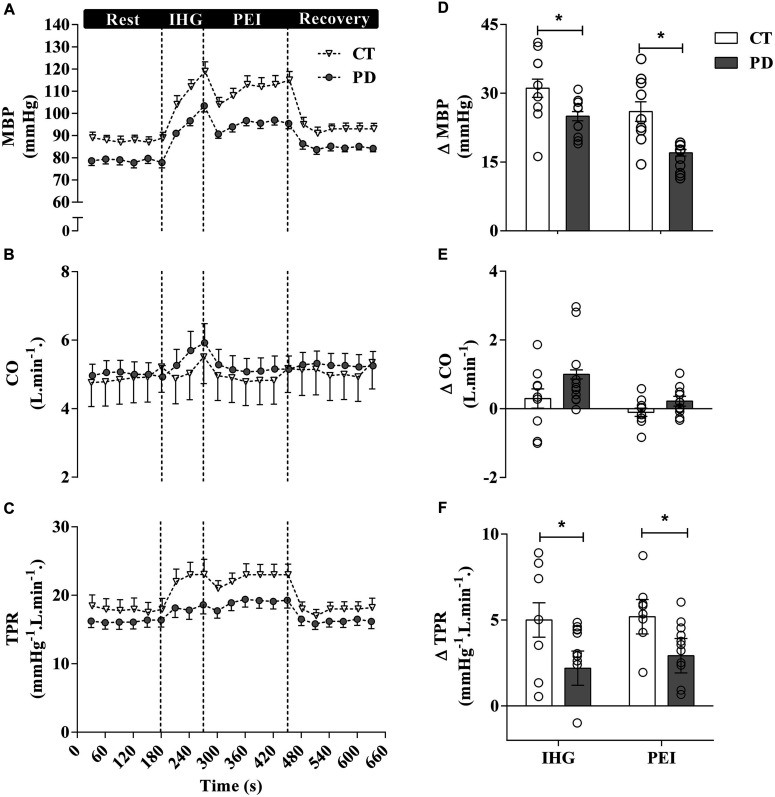
Mean and individual data from Sabino-Carvalho et al. (2018) demonstrated that mean BP (MBP) responses to exercise and post-exercise ischemia (PEI) are attenuated in patients with PD compared to healthy control (CT) subjects. These blunted cardiovascular responses to isometric handgrip exercise in patients with PD are partially attributable to an altered metaboreflex. CO, Cardiac output; TPR, total peripheral resistance.

Sympatho-excitatory mechanisms are activated during exercise (e.g., exercise pressor reflex and central command), where both mechanically and metabolically sensitive sensory fibers provide feedback via the dorsal horn of the spinal cord to brainstem cardiovascular areas in response to mechanical (i.e., mechanoreflex) and metabolic stimuli (i.e., metaboreflex), respectively (Coote et al., 1971; McCloskey and Mitchell, 1972; Teixeira et al., 2020). The latter has a major role in regulating the sympathetically mediated increases in cardiac contractility, stroke volume, heart rate, peripheral resistance and, consequently, BP during exercise (Fisher et al., 2015; Teixeira et al., 2018). Sabino-Carvalho et al. (2018) assessed the contribution of the metabolic component of the exercise pressor reflex on cardiovascular responses to isometric handgrip exercise in patients with PD, using a experimentally approach described in a landmark study by Alam and Smirk (1937). As indicated in Figure 3, following isometric handgrip exercise performed at 40% of maximum voluntary contraction, a period of post-exercise ischemia was applied in the exercising arm to trap the metabolites generated during muscle contraction. The increases in BP were blunted during exercise and during the isolation of muscle metaboreflex in patients with PD when compared to control subjects. Responses to a non-exercise sympatoexitatory maneuver (i.e., the cold pressor test) did not differ between groups, which suggest no group differences in generalized sympathetic responsiveness. Therefore, this study suggests that attenuated BP responses to exercise observed in PD are partially explained by an altered metaboreflex in regulating the sympathetically mediated cardiovascular responses during exercise. However, this response does not exclude the involvement of a parasympathetic dysfunction in this altered response.

Parasympathetic activation produces negative inotropic, chronotropic and dromotropic effects on the heart (Armour, 2011; Coote, 2013). The activity of the DMV vagal preganglionic neurons is responsible for tonic parasympathetic control of ventricular excitability (Machhada et al., 2015, 2016, 2017). Machhada et al. demonstrated that silencing of DMV vagal preganglionic neurons provide parasympathetic control of left ventricular inotropy (Machhada et al., 2016), and that decreased activity of DMV provides a neurophysiological basis for the progressive decline of exercise capacity with aging and in disease states (Machhada et al., 2017). By using an insect peptide (allatostatin) to inhibit DMV neurons the authors were able to show a dramatic reduction in exercise capacity. Noteworthy, along with this blunted exercise capacity, peak BP and heart rate during exercise were also blunted with DMV silencing, while DMV optogenetic recruitment enhanced the cardiac contractility and prolonged the exercise capacity (Machhada et al., 2017). Taken together, these results suggest that intact vagal activity generated by the DMV is required for proper cardiovascular adjustment to exercise.

Lowered vagal activity generated by the DMV might play a role in the attenuated cardiovascular responses, as well as, lowered exercise capacity presented by some patients with PD (Kanegusuku et al., 2016; Sabino-Carvalho et al., 2018). In an animal model of PD, Machhada et al. (2015) demonstrated that PD leads to a markedly reduction in DMV activity and this reduced activity led to changes in the electrophysiological properties of the ventricles, with a significantly shorter right ventricular effective refractory period than control animals and a QT prolongation. The attenuation was specifically in older animals (reflecting the PD progression), and that this reduced DMV neuron activity produced similar changes in ventricular excitability to that observed in control animals after DMV inhibition. Therefore, considering that the DMV neurons are affected by the pathological progression of PD (Braak et al., 2003) and intact DMV parasympathetic activity seems to determine cardiac contractility and exercise capacity, in animal model (Machhada et al., 2017), this raises the hypothesis that DMV vagal preganglionic neurons might be also involved in the blunted cardiovascular responses to exercise, as well as, the exercise capacity observed in patients with PD. However, further studies are needed to explore this hypothesis.

### Exercise Training in PD

There is currently no cure for PD, and although the primary resource for symptomatic control is pharmacologic-based, non-pharmacological low-cost approaches, such as exercise training, are being thought of as the universal prescription for PD (Alberts and Rosenfeldt, 2020; Lavin et al., 2020). Epidemiologic evidence suggest that moderate to vigorous exercise training might be protective against the developing of PD (Xu et al., 2010) and the growing body of evidence supports the beneficial effects of exercise on both motor (Silva-Batista et al., 2020a) and non-motor (Ganesan et al., 2014) symptoms. Furthermore, positive results regarding exercise training has been shown in PD animal models (Zhou et al., 2017). In addition to these positive effects, exercise has been employed as a coping resource to manage the quality of life (Corcos et al., 2013) and sleep (Silva-Batista et al., 2017), motor function (David et al., 2016; Vieira-Yano et al., 2020; Silva-Batista et al., 2020b), physical capacity (Kelly et al., 2014), fatigue (Santos et al., 2016) of patients with PD, and disease progression in a PD animal model (Zhou et al., 2017). Despite this, exercise training could be a challenging approach in this population, especially in those patients with OH (Roberson et al., 2019; Sabino-Carvalho et al., 2019; Sabino-Carvalho and Vianna, 2020), because of the impaired autonomic function (Goldstein, 2014; Vianna et al., 2016).

## Conclusion

Optimal autonomic nervous system function is crucial for evoking appropriate cardiovascular and hemodynamic adjustments to exercise. Emerging evidence suggests that cardiac parasympathetic activity may be also a determinant of exercise capacity. In PD, the pathogenesis affects both parasympathetic and sympathetic nerve activity, which in turn seems to mediate the abnormal cardiovascular responses observed during exercise. These findings may be important in the understanding the complexity of the neural control of circulation in patients with PD. Further studies are needed to better understand the pathophysiological underpinnings on the exercise response in PD.

## Author Contributions

JS-C wrote the first draft of the manuscript. All the authors read and revised it critically for important intellectual content. All authors approved the final version of the manuscript.

## Conflict of Interest

The authors declare that the research was conducted in the absence of any commercial or financial relationships that could be construed as a potential conflict of interest.
